# Antidepressants in paediatric depression: do not look back in anger but around in awareness

**DOI:** 10.1192/bjb.2017.2

**Published:** 2018-02

**Authors:** David A. Brent, Robert D. Gibbons, Paul Wilkinson, Bernadka Dubicka

**Affiliations:** 1 Western Psychiatric Institute and Clinic, University of Pittsburgh Medical Center; 2 University of Pittsburgh School of Medicine; 3 The University of Chicago Biological Sciences; 4 University of Cambridge; 5 Cambridgeshire and Peterborough NHS Foundation Trust, Cambridge; 6 Institute of Brain, Behaviour and Mental Health, University of Manchester; 7 Lancashire Care Foundation Trust, Preston

## Abstract

**Summary:**

In this paper, we summarise and critique a network meta-analysis (NMA) of antidepressant efficacy and tolerability for paediatric depression and an accompanying editorial. Although we agree that many of the extant studies are flawed, this meta-analysis showed clear efficacy of fluoxetine in the NMA, and for sertraline and escitalopram in pairwise analyses. Consequently, these papers underestimate the benefits of antidepressants for paediatric depression, and provide support for current practice guideline, which recommends the use of an antidepressant if the patient does not respond to psychotherapy. In these circumstances, fluoxetine should be the first choice, with escitalopram and sertraline as alternatives.

**Declaration of interest:**

D.A.B. receives royalties from Guilford Press, has or will receive royalties from the electronic self-rated version of the C-SSRS from eResearch Technology, Inc., is on the editorial board of UpToDate, and is a reviewer for Healthwise. R.D.G. serves as an expert witness for the US Department of Justice, Pfizer, Wyeth and GSK; and is the founder of Adaptive Testing Technologies. P.W. receives personal fees from Lundbeck and Takeda. B.D. reports a licensing agreement with Lundbeck for a psychosocial treatment manual for depression. No other disclosures were reported.

In this article, we review a network meta-analysis (NMA) that examines the relative efficacy and tolerability of antidepressants for paediatric depression, and the accompanying editorial that appeared in the Lancet. The meta-analysis provides useful information for researchers and practitioners, but undervalues the benefits of antidepressants and over-weights the risks.^1^ The accompanying editorial takes this bias as its point of departure, fuelled by righteous indignation about the deceptive reporting and publication practices of some industry-led studies.^2^ While this anger is justified, we believe that it should not preclude the reporting of benefits from the use of antidepressants in depressed youth, when supported by data.

Cipriani and colleagues have contributed a carefully and transparently conducted NMA of the comparative efficacy and tolerability (defined as discontinuation of the drug) of antidepressants for paediatric major depression.^1^ This meta-analysis examined 34 randomised clinical trials (RCTs) that encompassed 5260 participants and tested 14 different antidepressants. In the overall NMA, fluoxetine was the only agent found to be significantly more effective than placebo in reducing depressive symptoms (*d*  =  −0.51, 95% CI −0.99 to −0.03). Fluoxetine was no different from placebo with respect to tolerability and suicidal events, and was better tolerated than duloxetine or imipramine. Furthermore, imipramine, venlafaxine and duloxetine had more discontinuations than placebo. The quality of evidence was rated as ‘very low’ for most comparisons. The authors concluded that antidepressants do not appear to offer a clear advantage for children and adolescents, but if a pharmacological intervention is indicated, fluoxetine is ‘probably the best option’.

Pairwise meta-analyses found that three agents, fluoxetine, sertraline and escitalopram, showed superiority to placebo for both continuous and dichotomous outcomes. Venlafaxine was reported to have a higher incidence of suicidal events (OR = 0.13, 95% CI 0.00–0.55) than placebo and several other antidepressants, whereas none of the other agents studied had a higher rate of suicidal events than placebo.

The authors are to be commended for their careful attention to methodological quality, use of NMA and elegant presentation of results. We agree with the authors about the limitations imposed by low study quality, possible industry bias, incomplete assessment of suicidal events and overall small number of studies. This leads us to a different conclusion, which is that more studies that are conducted more rigorously are needed. Moreover, we found the interpretation of the findings to be inconsistent in three ways – with the data itself, across findings and compared with previous reports by some of the authors.

For example, the meta-analysis found that fluoxetine, compared with placebo, resulted in a medium effect size, yet because the confidence interval upper limit was close to zero (−0.99 to −0.03), the authors raise the question of ‘whether this estimate is robust enough to inform clinical practice’. Many statisticians would agree that after passing the muster of NMA, this effect is robust enough. The authors do not offer a bar above which these data are expected to pass. Moreover, the authors support the use of evidence-based psychotherapy for the treatment of paediatric depression, although it has a much smaller effect on paediatric depression than does fluoxetine (*d* = 0.29).^3^

The authors raised appropriate suspicion about studies conducted by industry, yet, in the case of fluoxetine, they raise the reverse concern that most of the fluoxetine trials were done *without* industry sponsorship and were of smaller sample size, ‘which might result in an exaggerated treatment effect’. These concerns are not grounded in facts. First, the National Institute of Mental Health (NIMH)-sponsored Treatment of Adolescent Depression Study (TADS) was one of the largest placebo-controlled paediatric depression trials, involving 423 young people.^4^ Second, since both published and unpublished studies with small samples were included, findings are just as likely to yield a Type II error as to result in a spurious positive finding. Third, other studies of paediatric and adult depression have found that the effect size is lower, and the placebo effect higher, when a greater number of sites is involved, as is more common in industry-sponsored studies.^5^^–^^7^ In fact, a paper co-authored by the lead author examining trends in clinical trials of antipsychotics observed that ‘effect sizes were reduced by industry sponsorship and increasing placebo response, not decreasing drug response’, and recommended that ‘drug development may benefit from *smaller samples but better-selected patients*’ (italics ours).^8^ We would argue that ‘smaller samples’ of ‘better-selected patients’ would be considered to be a positive design feature in antidepressant RCTs for paediatric depression as well. Finally, re-analyses of the fluoxetine trials for paediatric depression using patient-level data have found efficacy in the reduction of depression comparable to that reported in adults.^9^

The authors found that both sertraline and escitalopram had significant effects on the reduction in depressive symptoms on pairwise meta-analyses that apparently did not survive NMA. Given the reported high degree of homogeneity in these studies, and the fact that there were no indirect comparisons that would contribute extra studies, NMA may be inappropriately stringent. In the case of low heterogeneity, fixed effects meta-analysis, with narrow confidence intervals, may be more appropriate than the random effects used in NMA. Moreover, it may be justified to report also on the effects of *antidepressant drug classes* such as selective serotonin reuptake inhibitors (SSRIs), serotonin–noradrenaline reuptake inhibitors (SNRIs) and tricyclic antidepressants (TCAs). Inspection of the data suggests that SSRIs would show a more favourable effect on depressive symptoms and better tolerance compared with TCAs, which is a message found in previous meta-analyses, but worth repeating.^10^

The authors state that the risk–benefit profile is not favourable for antidepressants. Part of this argument is based on wide confidence intervals for efficacy. It seems inconsistent then to not put similar emphasis on the equally wide confidence intervals for discontinuation and suicidality, especially as the antidepressants with the best evidence for efficacy (i.e., fluoxetine, sertraline and escitalopram) are not significantly different from placebo for discontinuation or suicidality. With respect to suicidality, the rates of suicidal events ranged from 0 to 13% in those assigned to the drug, and 0 to 14% for those assigned to placebo. The authors report on a strong association between venlafaxine and suicidal events (OR = 0.13, 95% CI 0.00–0.55). While the rate of suicidal events in those assigned to venlafaxine (4%) was statistically higher than in those assigned to placebo (0%), the absence of events in the placebo group makes it difficult to obtain an accurate estimate of the odds of suicidal events associated with this agent.

The authors correctly point out limitations in the design and conduct of these clinical trials, but there are other limitations that greater access to data cannot remedy. First, the assessment of suicidal events in the majority of these studies was based on spontaneous report, rather than systematic assessment. One study that compared the rate of events by spontaneous reporting and systematic assessment found that spontaneous reporting of suicidal events underestimated the rate of events by a factor of 2.5.^11^ Moreover, patients on medication, perhaps owing to side-effects, might be more likely to report suicidal events, thus biasing conclusions based on these methods. In one of the few placebo-controlled trials that utilised systematic assessment of suicidal events, no difference was found between duloxetine, fluoxetine and placebo in the frequency of suicidal events.^12^ Second, there are inherent limitations in clinical trials, since those patients most likely to be treated with an antidepressant are least likely to be enrolled into an RCT. For example, a recent suicide attempt is an exclusion for almost all paediatric depression pharmacotherapy RCTs. In a study of the relationship between a suicide attempt and initiation of antidepressant treatment in one large healthcare system, a suicide attempt was a common precipitant for *starting* an antidepressant in adolescents and in adults.^13^ Moreover, in these youth, the rate of suicide attempts was highest *prior* to the initiation of treatment, suggesting that antidepressants are protective against suicidal behaviour, even in young people. Although causal inferences cannot be firmly drawn from observational studies, such studies have the advantage of larger size, representativeness, longer duration of treatment, and ability to link treatment to suicide, not just to suicidal events. While not incontrovertible, there are many observational studies showing strong inverse associations between prescription and sales rates of SSRIs and suicide, including suicide in adolescents.^14^^–^^16^ Conversely, after the Black Box Warning, there has been at least a temporary decline in antidepressant prescriptions in the United States, Canada and The Netherlands, accompanied by an uptick in adolescent suicides.^17^^,^^18^ If antidepressants were strongly associated with suicide, one would expect that a decline in antidepressant prescriptions would be accompanied by a corresponding decline in suicide, rather than the exact opposite.

This carefully conducted meta-analysis was accompanied by an editorial that was a polemic against the use of antidepressants, entitled ‘Antidepressants fail, but no cause for therapeutic gloom’.^2^ The editorialist asserted that the extant literature greatly exaggerates the benefits of antidepressants and downplays their risks, owing to the poor data quality and selective reporting of results. He recommended that clinicians reading the literature assume that the benefits of a drug are inflated, and that the occurrence of harmful events is more serious and frequent than reported. This editorial asserted that the reported association between fluoxetine treatment and improvement in symptoms and functional outcomes is not necessarily causative, and that fluoxetine is likely to be more dangerous, and less effective than presented in the extant literature. This editorial further opined that fluoxetine has never been compared with a supportive relationship, which the editorialist considered was likely to be more helpful and less harmful than antidepressant medication for depressed youth. The editorial concluded that industry-sponsored research should provide transparency and access to all data and procedures.

It is only with the last statement in this editorial that we can proffer agreement. We wholeheartedly endorse the need for data transparency in all clinical trials, including those conducted by industry, and acknowledge the damage to the credibility of all studies caused by failure to publish and disclose data. However, the largest single clinical trial of antidepressants in paediatric depression, TADS, was not sponsored by industry, but by NIMH. This study showed a higher rate of response and better functioning in those assigned to fluoxetine versus those assigned to placebo.^4^^,^^19^ While the editorialist raised the question of whether association implies causality, a blinded placebo-controlled trial has long been considered the gold standard for causal inferences. The editorialist also suggests that supportive therapy is likely to be more effective, and less harmful, than fluoxetine. However, the placebo condition in TADS involved supportive management, and resulted in a response rate of only 35% *v.* 61% for those treated with fluoxetine.^4^ Although there were more spontaneously reported suicidal events (which includes thoughts without acts) in those treated with fluoxetine than in those treated with placebo, the response rate after 12 weeks of treatment was much higher in those treated with fluoxetine, and absolute risk differences were several-fold higher for efficacy than for suicidal events.^20^^,^^21^ Moreover, a comparison of another version of cognitive–behavioural therapy (CBT) with supportive treatment found that CBT was much more efficacious.^22^ Therefore, results of the TADS trial and other published data completely refute the editorialist's assertions that industry sponsorship of some studies automatically dilutes the efficacy reports of medication, including fluoxetine, that causality cannot be inferred from an RCT, and that supportive care would be superior to treatment with fluoxetine.

What is a clinician to make of this meta-analysis and editorial? We believe that the findings from the meta-analysis support current clinical guidelines. In the UK, National Institute for Health and Care Excellence (NICE) guidelines advise the first-line use of an evidence-based psychotherapy such as interpersonal therapy or CBT, and, if the patient does not respond, to then consider adding pharmacotherapy, namely, fluoxetine.^23^ We continue to support this approach. Also, as per NICE guidelines, in cases of severe, chronic or treatment-resistant depression, there is evidence to support starting with a combination of psychotherapy and pharmacotherapy, which has been shown to be superior to medication monotherapy for treatment-resistant depression.^23^^–^^25^ Despite the 0% rate of suicidal events in the placebo cells for venlafaxine studies, we agree that caution is indicated in the use of this agent, insofar as SSRIs are just as effective for treatment-resistant depression as venlafaxine, but their use results in fewer side-effects, and lower levels of suicidal ideation and events.^11^^,^^26^ Moreover, these meta-analyses are consistent with the view that other antidepressants, such as sertraline and escitalopram, are reasonable and effective alternatives should patients have a history of not responding to an adequate trial of fluoxetine. The risk–benefit ratio for use of antidepressants in paediatric depression is relatively favourable, with about 11 times more young people responding to an antidepressant than developing suicidal events.^5^ Moreover, the shadow cast by the Black Box Warning should not discourage the clinician from using antidepressants for the treatment of anxiety and obsessive–compulsive disorders, as treatment of these conditions with SSRIs is more likely to result in a clinical response, and less likely to result in a suicidal event, compared with antidepressant treatment of paediatric depression.^5^^,^^27^

Our job as responsible scientists and clinicians is to inform patients and families about the risks and benefits of each intervention, with appropriate confidence intervals and without bias, and to use this information to collaborate with families in making clinically appropriate treatment decisions. It is justifiable to be angry about scientific obfuscation and deception, but we should not paint all studies and findings with the same broad brush. Instead, let us consider the wise words of the blind, but insightful, author James Thurber, who advised us to ‘not look back in anger, or forward in fear, but around in awareness’.^28^

## About the authors

David A. Brent MD, Western Psychiatric Institute and Clinic, University of Pittsburgh Medical Center, Pittsburgh, PA, and Department of Psychiatry, University of Pittsburgh School of Medicine, Pittsburgh, PA; Robert D. Gibbons, PhD, Departments of Medicine and Public Health Sciences, The University of Chicago Biological Sciences, Chicago, IL; Paul Wilkinson, MD, Developmental of Psychiatry, University of Cambridge, and Cambridgeshire and Peterborough NHS Foundation Trust, Cambridge, UK; Bernadka Dubicka, MD, Institute of Brain, Behaviour and Mental Health, University of Manchester, UK, and Lancashire Care Foundation Trust, Preston, UK.
